# Visual function changes in a Parkinson’s Disease cohort – preliminary data

**DOI:** 10.22336/rjo.2021.48

**Published:** 2021

**Authors:** Vlad-Ioan Suciu, Corina-Iuliana Suciu, Simona Delia Nicoară, Lăcrămioara Perju-Dumbravă

**Affiliations:** * Department of Neuroscience, “Iuliu Haţieganu” University of Medicine and Pharmacy, Cluj-Napoca, Romania; ** University of Oradea, Romania; *** Department of Ophthalmology, “Iuliu Haţieganu” University of Medicine and Pharmacy, Cluj-Napoca, Romania

**Keywords:** epidemiology, Parkinson’s disease, Transylvania region, study sample characteristics, quality of life

## Abstract

The prevalence of Parkinson’s disease (PD), the second most common neurodegenerative disorder, has dramatically increased worldwide from 2,5 million in 1990, to 6,1 million in 2016. This condition continues to unfold its complexity, being nowadays recognized more as a syndrome rather than a disease. Beside the motor symptoms, the non-motor features, which can appear as early as twenty years before the classic onset, are now included in the diagnostic criteria.

Increased public awareness, early recognition, and intervention (mostly neuroprotective) could highly increase the quality of life of people suffering from PD. We believe that these measures must be taken without delay, in order to counteract the increasing prevalence of PD worldwide.

**Abbreviations:** PD = Parkinson’s disease, GBD = Global Burden of Diseases, HY = Hoehn-Yahr scale, SD = standard deviation

## Introduction

Parkinson’s disease (PD) is the second most prevalent neurodegenerative disorder worldwide [**1**-**3**].

According to the GBD 2016 Parkinson’s Disease Collaborators, the prevalence of PD increased dramatically worldwide from 2,5 million (in 1990) to 6,1 million (in 2016). The risk factors are advanced age, industrial chemicals, and pollutants (pesticides, solvents, metals). Protective factors are smoking, coffee and others [**4**].

The clinical diagnosis accuracy is about 80-90%. Non-motor features appearing in the prodromal phase can be present 20 years before the onset of the classic motor features of PD [**2**]. The non-motor features, being recognized today more frequently, are now included in the supportive criteria for diagnosing PD. The visual function changes are considered among the non-motor features in the clinical picture [**5**,**6**].

The aim of this paper was to describe the characteristics of a PD study sample recruited from Transylvania Region, Romania, and to compare our findings with the current literature. This article is the first part of the analysis of this sample.

## Materials and methods

We report the first part of the analysis of a Parkinson’s disease (PD) study sample. This was an observational, prospective study, performed in Cluj, Transylvania, Romania. All patients were recruited from the Neurology Clinic I of the County Emergency Hospital Cluj-Napoca, Romania. Upon recruitment, all patients signed an informed consent and met the inclusion criteria. Only adult PD patients (>18 years) were included in this study. Advanced stages, PD related complications, psychiatric or previous ocular pathologies constituted exclusion criteria. This study adhered to the Declaration of Helsinki. Statistical significance was considered for p<0.05.

## Results

Parkinson’s disease patients were recruited from the County Emergency Hospital Cluj-Napoca, Romania. The inclusion criteria were adult patients diagnosed with Parkinson’s disease, stages 1-3 Hoehn-Yahr (HY), with no prior history of severe ocular pathology, psychiatric conditions or severe complications related to PD. All patients enrolled in this study signed a board approved informed consent. A cohort of twenty-five PD patients were included to date. Only 4% had a positive family history of PD. All subjects had normal intraocular pressure measured by aplanotonometry.

An unequal gender distribution was found, with 32% of the patients being women and 68% men. The mean age was 67.36 ± 8.2 (SD), ranging from 49 to 85 years.

**Fig. 1:**
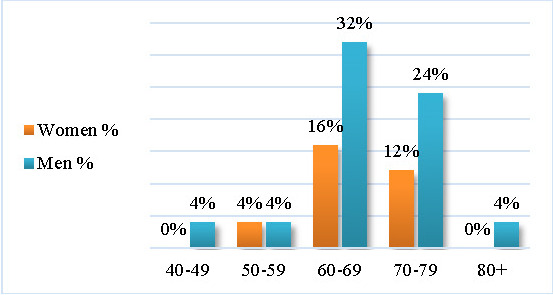
The distribution by age and gender

The distribution of patients according to age and gender revealed that most women (16% of the total sample) were in the 60-69 years’ age group, while no woman was in the 40-49 years and 80+ age groups. Most men (32%) were in the 60-69 years’ age group. Both genders had the highest incidence in the 60-69 years and 70-79 years’ age group (see **Fig. 1**).

Most recruited patients came from urban areas (92%) versus rural areas (8%) (see **Table 1**).

**Fig. 2:**
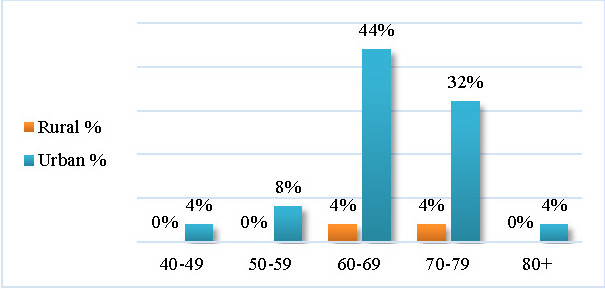
The distribution by age group and geographical area

The graph showing the distribution of the enrolled patients by age group and geographical area revealed that most urban inhabitants were in the 60-69 years (44%) and 70-79 years (32%) age group. Moreover, rural inhabitants were found only in these two age groups (see **Fig. 2**).

92% of the individuals retired from activity (see **Table 1**).

**Fig. 3:**
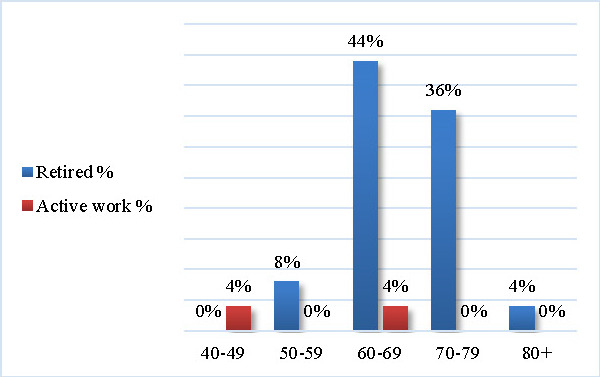
Professional activity status by age groups

Regarding the status of the professional activity, the age group with the most retired individuals (44%) was the 60-69 years’ age group. On the other hand, no retired patient was found in the 40-49 years’ age group (see **Fig. 3**).

The mean disease duration in years was 9.29 ± 6.04 (SD) for this sample. Regarding the educational level, 60% of the patients had university-level education (see **Table 1**).

**Tabel 1:** Characteristics of the study sample

Characteristic	PD Sample
Women %	32
Men %	68
Age, years: Mean ± SD	67.36 ± 8.2
Disease duration, years: Mean ± SD	9.29 ± 6.04
Urban area %	92
Rural area %	8
Retired %	92
University education %	60

 The analysis of the study sample based on the Hoehn-Yahr grading scale revealed that 8% of the PD patients were classified within the first stage of evolution, while the most (36%) were classified in the third stage of evolution. Moreover, the HY stages 2, 2.5 and 3 had similar distributions of individuals. Regarding the gender distribution, no woman was classified in the first HY stage, while most women (20%) were classified in the second HY stage. The fewest men (8%) were classified in the first HY stage, while the most (28%) were classified in the third HY stage (see **Table 2**).

The MDS-UPDRS score Part III was analyzed in the “On” Phase for all individuals and showed an increasing mean total score from HY stages 1 to 3: 16.5 ± 0.7 (SD) for the first HY Stage; 24.75 ± 7.14 (SD) for the second HY Stage; 27.5 ± 7.17 (SD) for the HY Stage 2.5 and 47.55 ± 8.11 (SD) for the third HY Stage (see **Table 2**).

The ANOVA statistical analysis revealed highly significant differences (p<0,0001) between the MDS-UPDRS Part III scores and the HY Stage-groups.

The shortest mean disease duration measured in years was 3.82 ± 2.84 (SD) for the first HY Stage, while the longest disease duration was 13.77 ± 5.75 (SD) for the third HY Stage (see **Table 2**).

The ANOVA statistical analysis showed highly significant differences (p<0.01) between the disease duration (years) and the HY Stage-groups.

**Tabel 2:** Characteristics of PD patients based on Hoehn-Yahr stage

	HY Stage 1	HY Stage 2	HY Stage 2.5	HY Stage 3
% of PD Patients	8%	32%	24%	36%
% Women	0%	20%	4%	8%
% Men	8%	12%	20%	28%
MDS-UPDRS Part III On: Mean ± SD	16.5 ± 0.7	24.75 ± 7.14	27.5 ± 7.17	47.55 ± 8.11
Disease Duration (Years) Mean ± SD	3.82 ± 2.84	8.98 ± 5.71	4.78 ± 1.41	13.77 ± 5.75
*ANOVA MDS-UPDRS Part III differences between HY groups (p<0.0001);				
*ANOVA Disease duration differences between HY groups (p<0.01).				

**Fig. 4:**
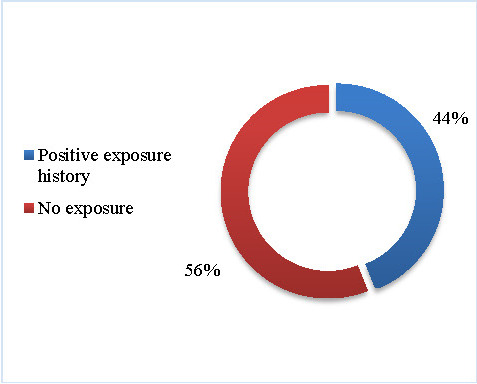
Professional toxic exposure

56% of the individuals reported no professional toxic exposure (see **Fig. 4**).

The most prevalent PD subtype was found to be the tremor predominant type (60%), while the least prevalent subtype was the equivalent type (4%).

**Fig. 5:**
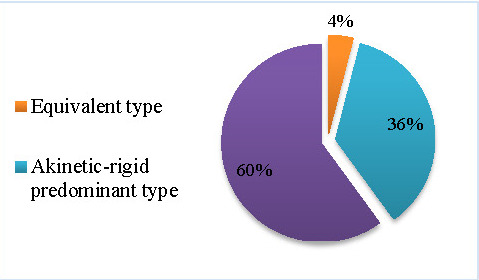
PD subtypes in this study sample

**Fig. 6:**
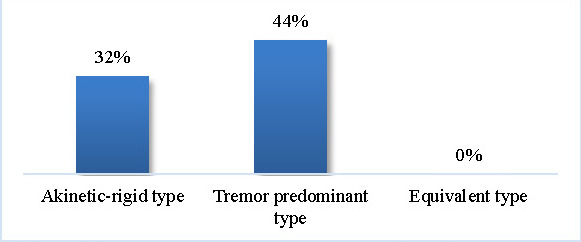
Visual symptoms according to the PD subtypes

76% reported at least one visual symptom (blurry vision, dry eye syndrome, etc.). The tremor predominant type was most correlated with the appearance of visual symptoms (44%) (see **Fig. 6**).

**Fig. 7:**
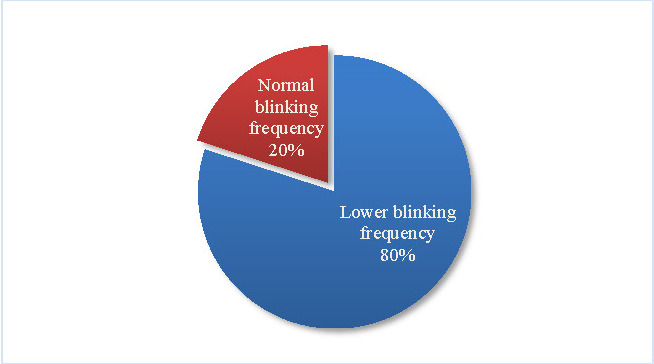
Blinking frequency

Most PD patients (80%) displayed a reduced blinking frequency (see **Fig. 7**).

**Fig. 8:**
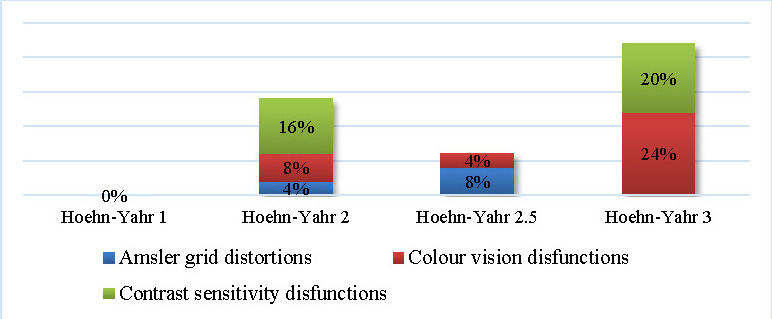
Visual function changes correlated with the Hoehn-Yahr stages

84% of the entire PD sample demonstrated some degree of visual impairment when tested with the Amsler grid, Ishihara, and Pelli-Robson test. According to the Hoehn-Yahr PD severity scale, the analysis of the visual function changes revealed no visual changes in the first stage of evolution. The color vision (24%) and contrast sensitivity (20%) dysfunctions are both predominantly found in the third H-Y stage (see **Fig. 8**).

**Fig. 9:**
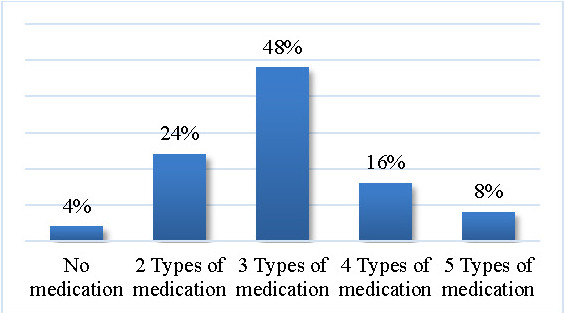
Parkinson’s disease medication complexity

48% of the entire sample had a PD medication plan consisting of 3 different types of molecules, while only 4% had no dopaminergic medication (see **Fig. 9**).

**Fig. 10:**
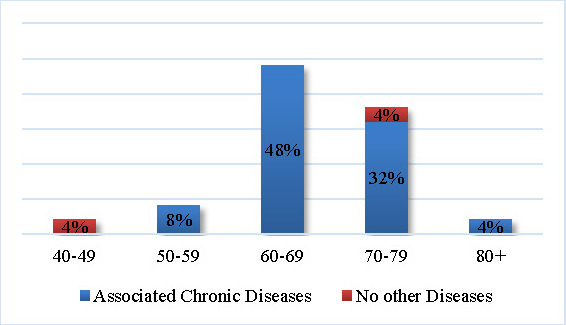
Comorbidities by age group

The graphical analysis of the comorbidities by age groups revealed that the most affected was the 60-69 years’ age group (48%). On the other hand, the 40-49 years’ age group reported no comorbidities (see **Fig. 10**).

## Discussions

According to Ascherio and Schwarzschild, 90% of PD patients have no genetic cause [**2**]. Other authors confirm that 5-10% of patients have genetic factors discovered during the investigations [**5**]. Our study showed that only 4% of the recruited PD individuals had a positive family history of PD and probably a genetic cause.

The lifetime risk for developing PD is greater in men (2%) versus women (1,3%) [**2**]. We found that the gender distribution in the study sample was unequal: 32% women and 68% men (1:2,1 = W:M), with the mean age of 67.36 years ± 8.2 (SD), ranging from 49 to 85 years. According to Tusnes and Storstein, PD prevalence increases with advancing age [**5**].

The yearly incidence of PD is 14/ 100.000 for the total population and 160/ 100.000 for individuals over 65 years old [**2**].

According to the current literature, the usual disease onset is between 40-70 years, while the mean age at onset is 60 years [**1**,**7**-**9**]. Our study has shown that the mean age for this sample was 67,36 years, with ages ranging from 49 to 85 years.

Europe has similar incidence rates like America and Asia, but greater than Africa. Hispanic and Afro-American individuals seem to have a higher risk for PD than Caucasian individuals [**2**]. Being performed in Cluj, all the participants in our study were Caucasian.

Some authors stated that rural living combined with agriculture and well-water consumption may be linked to PD [**10**]. However, this theory is difficult to be evaluated because rural living does not necessarily imply well-water consumption or the use of pesticides for agriculture. Also, the dosage and time of exposure to these potential risk factors is impossible to measure. Our epidemiologic study revealed that only 8% of the recruited individuals came from rural areas, making this potential risk factor insignificant.

In a working-aged study sample performed in Finland, 37% of the participants retired early, due to PD disability. The median age of retirement in this study was 53,4 years, being approximately 6 years earlier than the general population, according to Martikainen and colleagues [**11**]. In this present study, we showed that 92% of the enrolled patients retired from activity, while 8% retired in the 50-59 years’ age group and no retired person was found in the 40-49 years’ age group. Moreover, the mean disease duration in years was 9.29 ± 6.04 (SD). These findings could be explained by the fact that only patients with mild to moderate disease severity were included in this study.

Risk factors for developing PD seemed to be pesticides, dairy products, melanoma, traumatic brain injuries, on the other hand, protective factors were nicotine, caffeine, urate, physical activity, Ibuprofen and Calcium channel blockers [**2**]. We found that 56% of the recruited individuals reported no professional toxic exposure. Other risk or protective factors were difficult to confirm and quantify based on the patient history.

According to Rajput et al. (2009), in their study on 166 PD patients along 39 years of evolution (1968-2006), 66% were classified in the mixed PD subtype, 26% in the akinetic-rigid subtype and only 8% in the tremor dominant subtype. Moreover, the clinical evolution was found to be most favorable in the tremor-dominant subtype, which was followed by the mixed and then the akinetic-rigid subtypes [**12**]. In our study, however, we found that the most prevalent PD subtype was the tremor predominant type (60%), while the least prevalent subtype was the equivalent (or mixed) type (4%).

According to Csoti et al. (2016), there are 3 types of associated conditions to PD: independent comorbidities, conditions secondary to autonomic denervation and side effects of PD medication. Because of this complexity, PD should be monitored and approached multidisciplinary. In one study cited by them, 80% of PD patients had 5 or more associated diseases. Also, the most frequent associations were pathologies involving locomotion (joint pathologies and fractures). Cardiovascular and respiratory pathologies have high rates of mortality among PD patients [**13**]. In one review, Potashkin and colleagues showed that the cardiovascular risk factors and PD have intertwined pathological mechanisms. Therefore, genetic, and environmental factors both contribute to the development of dysregulation of several pathways such as glucose metabolism, cellular stress, lipid metabolism and inflammation. These pathways pave the way to the genesis of both PD and cardiovascular diseases [**14**].

We showed that the highest incidence of associated diseases was found in the 60-69 years’ age group (48%). Regarding the analysis of comorbidities that affect locomotion, over 3 comorbidities were found only in the 70-79 years’ age group (12% of the total sample). The analysis of the global cardiovascular risk showed that the 60-69 years and 70-79 years’ age groups had equal distributions (24%) of very high risk (≥ 3 risk factors present in the same individual).

A recent study suggests the correlation between the dopaminergic nigrostriatal loss and the structural changes of the retinal layers in PD. Furthermore, it is believed that the retina is affected by the pathogenic mechanisms before the development of the motor disease (parkinsonism). Also, the contrast sensitivity impairment could be the clinical validation of the dopaminergic loss in the retina [**15**]. Our study revealed the visual function impairments in a PD cohort. Even though 76% of the patients reported visual symptoms, when objectively tested, 84% had some degree of visual impairment. These impairments can be related to PD because all the enrolled participants were first screened for previous ocular pathologies, which could have affected the results. These clinical findings could hold a key for diagnosing PD more early with a good collaboration between neurologists and ophthalmologists.

## Conclusions

In this first report of the epidemiology and characteristics of a PD study sample recruited from Transylvania Region, Romania, we showed similarities with the cited literature. As an increasingly prevalent condition, PD needs to be recognized as early as possible in order to delay the neurodegeneration. Increased awareness, early recognition of the prodromal symptoms and a multidisciplinary approach are some of the mandatory actions that need to be taken without delay in order to increase the quality of life for our patients.

**Conflict of Interest statement**

The authors state no conflict of interest.

**Informed Consent and Human and Animal Rights statement**

Informed consent has been obtained from all individuals included in this study.

**Authorization for the use of human subjects**

Ethical approval: The research related to human use complies with all the relevant national regulations, institutional policies, is in accordance with the tenets of the Helsinki Declaration, and has been approved by the review board of “Iuliu Haţieganu” University of Medicine and Pharmacy, Cluj-Napoca, Romania.

**Acknowledgements**

All authors contributed substantially for this manuscript.

The authors acknowledge the technical support from “Iuliu Haţieganu” University of Medicine and Pharmacy Cluj-Napoca, Romania with the Departments of Neuroscience and Ophthalmology, and the County Emergency Hospital Cluj-Napoca, Romania.

We would also like to thank all the subjects who participated in this study.

**Sources of Funding**

No source of funding to declare.

**Disclosures**

None.
